# Cosmological analogies, Lagrangians, and symmetries for convective–radiative heat transfer

**DOI:** 10.1140/epjc/s10052-020-8292-0

**Published:** 2020-08-06

**Authors:** Valerio Faraoni, Farah Atieh, Steve Dussault

**Affiliations:** Department of Physics and Astronomy, Bishop’s University, 2600 College Street, Sherbrooke, Quebec J1M 1Z7 Canada

## Abstract

A formal analogy between the Friedmann equation of relativistic cosmology and models of convective–radiative cooling/heating of a body (including Newton’s, Dulong–Petit’s, Newton–Stefan’s laws, and a generalization) is discussed. The analogy highlights Lagrangians, symmetries, and mathematical properties of the solutions of these cooling laws.

## Introduction

Newton announced his model of cooling in 1701, with the rate of heat loss by convection from a body of area *A* at temperature *T* given by^1^

1 $$\begin{aligned} \frac{1}{A} \frac{dQ}{dt} = - h \left( T-T_{\infty } \right) , \end{aligned}$$

where $T_\infty$ is the temperature of the surroundings and *h* is a convection coefficient, assumed to be constant. Newton’s law of cooling has its limitations: it describes the cooling process well only for moderate temperature differences $\theta (t) \equiv T(t)-T_{\infty }$ and it works better for forced convection than for natural convection (see Ref. [1] for a pedagogical review).

By introducing the heat capacity *C* of the body, the elementary heat transferred in a time *dt* is $dQ=CdT$ and one obtains

2 $$\begin{aligned} \frac{dT}{dt} = -k \left( T-T_\infty \right) \end{aligned}$$

with $k \equiv A h/C$. It is mathematically convenient to use the temperature difference $\theta (t)$, in terms of which $\dot{\theta }=-k\theta$. Assuming that the surroundings have a much larger heat capacity than the cooling body, $T_{\infty }$ is constant and the solution of Eq. (2) is then

3 $$\begin{aligned} T(t) = \left( T_0 -T_{\infty }\right) \text{ e}^{-kt} + T_\infty \,, \end{aligned}$$

where $T_0=T(0)$ is the initial temperature. The final state is one of thermal equilibrium $T(t) \simeq T_{\infty }$, irrespective of the initial condition $T_0$.

Other models of convective cooling are used to describe regimes in which the temperature difference $\theta$ is large, including the 1817 Dulong–Petit law [2]

4 $$\begin{aligned} \frac{1}{A} \frac{dQ}{dt} = - g (T-T_a)^n \,, \end{aligned}$$

where the exponent *n* ranges between 1.25 and 1.6 [1, 3–5], which translates into

5 $$\begin{aligned} \frac{d\theta }{dt} = - g_0 \, \theta ^n \end{aligned}$$

($g_0 \equiv gA/C$). The solution is

6 $$\begin{aligned} \theta (t)= \frac{\theta _0}{ |t-t_0|^{\frac{1}{n-1}} } \,, \end{aligned}$$

where $\theta _0= \left[ (n-1)g_0\right] ^{\frac{1}{1-n}}$ and $t_0$ is an integration constant. This solution becomes singular as $t\rightarrow t_0$, hence it cannot be extended arbitrarily in the past.

Cooling by radiation instead is described (with better accuracy) by the Stefan–Boltzmann law. In practice, convective Newton cooling and radiative cooling often occur together, resulting in the Newton-Stefan cooling law

7 $$\begin{aligned} \frac{1}{A} \frac{dQ}{dt} = - h \left( T-T_\infty \right) - \epsilon \sigma \left( T^4 - T^4_{\infty }\right) \,, \end{aligned}$$

where $\sigma$ is the Stefan-Boltzmann constant and $\epsilon$ is the emissivity. Now the temperature difference evolves according to

8 $$\begin{aligned} \frac{d \theta }{dt} =-\left( \alpha \theta +\beta \theta ^2 + \gamma \theta ^3 + \delta \theta ^4 \right) , \end{aligned}$$

where

9 $$\begin{aligned} \alpha= & {} \frac{A}{C} \left( h + 4\epsilon \sigma T_{\infty }^3\right) \end{aligned}$$

10 $$\begin{aligned} \beta= & {} \frac{6A}{C} \, \epsilon \, \sigma T^2_{\infty } \end{aligned}$$

11 $$\begin{aligned} \gamma= & {} \frac{ 4A\epsilon \sigma T_{\infty }}{C} \,, \end{aligned}$$

12 $$\begin{aligned} \delta= & {} \frac{A \epsilon \sigma }{C} \,. \end{aligned}$$

A physically meaningful approximation occurs when the temperature difference $\theta$ is small and the higher order terms in $\theta$ can be dropped [1], reducing the evolution equation to

13 $$\begin{aligned} \frac{d\theta }{dt} =- \left( \alpha \theta + \beta \theta ^2\right) \,. \end{aligned}$$

This first order equation integrates to

14 $$\begin{aligned} \theta (t) = \frac{\theta _0 \, \text{ e}^{-\alpha \, t} }{1+ \Gamma \theta _0 \left( 1-\text{ e}^{-\alpha \, t}\right) }\,, \end{aligned}$$

where $\Gamma = \beta /\alpha$ and $\theta _0=\theta (0)$ is the initial temperature difference between the cooling body and its surroundings.

If one waits long enough, the exact Newton–Stefan cooling approaches the approximated model (13). In fact, a characteristic feature of spontaneous heat transfer is that temperature gradients decrease and temperature differences are smoothed out as thermal equilibrium is approached (which is the origin of the negative sign in the right-hand side of the cooling/heating model equations). Therefore, $\theta$ is bound to decrease to a size where higher order terms in the fourth order polynomial in $\theta$ in Eq. (8) become negligible and the evolution is then ruled only by the linear and quadratic terms. Waiting longer, only Newton cooling described by the linear term remains relevant. In other words, the decreasing exponential solution $\theta _0 \, \text{ e}^{-\alpha t}$ of Newton’s model is a late-time attractor in the phase space of the solutions of the full Newton–Stefan cooling equation (8), which ultimately justifies the approximated model (13).

There are formal analogies between these various cooling laws and spatially homogeneous and isotropic (or Friedmann–Lemaître-Robertson–Walker, “FLRW” in short) cosmology in the context of general relativity. We discuss these analogies after a brief summary of FLRW cosmology.

## FLRW cosmology

For the reader’s convenience, we recall here the basics of FLRW cosmology [6–9]. The most basic assumption is the Copernican principle stating that, on average (i.e., over scales larger than 300 megaparsecs) all spatial points and directions are equivalent: there are no preferred points and no preferred directions in space. The assumptions of spatial homogeneity and isotropy force the spacetime geometry to be the one described by the FLRW line element [6–8] given, in comoving polar coordinates $\left( t, r, \vartheta , \varphi \right)$, by^2^

15 $$\begin{aligned} ds^2= & {} -dt^2 +a^2(t) \left[ \frac{dr^2}{1-Kr^2} +r^2 \left( d\vartheta ^2 + \sin ^2 \vartheta \, d\varphi ^2 \right) \right] \,.\nonumber \\ \end{aligned}$$

The *scale factor* *a*(*t*) describes how two points of space at fixed comoving coordinate distance $$r_0$$ recede from each other with the cosmic expansion. Their physical distance at time *t* is $$ l(t)=a(t)r_0$$, increasing if $$ \dot{a}>0$$. The function *a*(*t*) maps the history of the cosmic universe, increasing in an expanding universe or decreasing in a contracting one.

The constant *K* (*curvature index*) in Eq. (15) describes, respectively, a closed universe (in which sections of constant time are 3-spheres) if $K>0$; or a universe with flat Euclidean spatial 3-sections if $K=0$; or a universe with hyperbolic 3-dimensional sections of constant time *t* if $K<0$ [6–9]. These are the only possible FLRW geometries and the dynamics is embodied by the evolution of the scale factor *a*(*t*).

In cosmology, the matter content of the universe, which causes the spacetime to curve, is usually modelled by a perfect fluid of energy density $\rho (t)$ and isotropic pressure *P*(*t*) related by some equation of state. Assuming the FLRW geometry, the Einstein field equations of general relativity reduce to the so-called Einstein–Friedmann equations for the functions $a(t), \rho (t)$, and *P*(*t*) [6–9]

16 $$\begin{aligned}&H^2 \equiv \left( \frac{\dot{a}}{a}\right) ^2 =\frac{\Lambda }{3} + \frac{8\pi G}{3} \, \rho -\frac{K}{a^2} \,, \end{aligned}$$

17 $$\begin{aligned}&\frac{\ddot{a}}{a}= -\, \frac{4\pi G}{3} \left( \rho +3P \right) -\frac{\Lambda }{3} \,, \end{aligned}$$

18 $$\begin{aligned}&\dot{\rho }+3H\left( P+\rho \right) =0 \,, \end{aligned}$$

where $\Lambda$ is Einstein’s cosmological constant, an overdot denotes differentiation with respect to *t*, and $H(t)\equiv \dot{a}/a$ is the Hubble function [6–9]. Out of these three equations, only two are independent. If any two are given, the third one can be derived from them. Without loss of generality, we take the Friedmann equation (16) and the energy conservation Eq. (18) as primary, with the acceleration Eq. (17) following from them.

In cosmology, it is common (although not compulsory) to assume that the cosmic fluid satisfies the barotropic equation of state

19 $$\begin{aligned} P=w\rho \end{aligned}$$

where the “equation of state parameter” $w=$ const. Then, Eq. (18) integrates to

20 $$\begin{aligned} \rho (a) = \frac{ \rho _0}{ a^{3(w+1)} } \end{aligned}$$

(independent of the curvature index *K*), where $\rho _0$ is a positive constant determined by the initial conditions. In a spatially flat universe, this gives

21 $$\begin{aligned} a(t) = a_0 \left( t-t_0 \right) ^{\frac{2}{3(w+1)} } (w\ne -1). \end{aligned}$$

We can already see how the analogy with cooling works: exchanging the variables $\left( t, \theta (t) \right) \longrightarrow \left( t, a(t) \right)$, the cooling equation is rewritten as $\dot{\theta }= \theta f(\theta )$ and squared, obtaining

22 $$\begin{aligned} \left( \frac{1}{\theta } \, \frac{d\theta }{dt} \right) ^2 = f^2(\theta )\,. \end{aligned}$$

The analogy carries through provided that a suitable cosmological fluid fills the analogous universe. This is obtained by imposing the second independent equation, *i.e.*, the energy conservation Eq. (18). By comparing Eqs. (16) and (22), we see that it must be

23 $$\begin{aligned} \frac{8\pi G}{3} \, \frac{ \rho _0}{ a^{3(w+1)} } =f^2(\theta )\,. \end{aligned}$$

This equation is satisfied if $f(\theta )$ is a power law or an inverse power law. One can also consider mixtures of non-interacting perfect fluids in which the energy densities $\rho _{(i)}$ and partial pressures $P_{(i)}$ of the individual fluids add up according to Dalton’s law. In the simpler case described by Eq. (23), provided that $f(\theta )$ has a suitable form, the analogy between cooling/heating and cosmology is established when the analogous universe is filled with a perfect fluid with $P=w\rho$.

A special solution is the de Sitter spacetime corresponding to a spatially flat ($K=0$), empty universe sourced by a positive cosmological constant $\Lambda$, which has equation of state parameter $w=-1$. The Friedmann equation (16) reduces to

24 $$\begin{aligned} H^2 = \frac{\Lambda }{3} = \, \text{ const. } \end{aligned}$$

and the solution is a simple exponential $a(t)=a_0 \, \text{ e}^{\pm H_{(\pm )}t}$ with constant Hubble function $H_{(\pm )}= \pm \sqrt{\Lambda /3}$.

In addition to the previous standard textbook material there are other, lesser known, aspects of FLRW cosmology in recent technical literature that unveil unknown features of the cooling laws. They are reported in the following subsections.

### Lagrangian and Hamiltonian

An effective Lagrangian for spatially flat FLRW cosmology (to which we will reduce in the following, although generalizations are possible) is

25 $$\begin{aligned} L\left( a, \dot{a} \right) = 3a\dot{a}^2 + 8\pi G a^3 \rho \,, \end{aligned}$$

where the function $\rho =\rho (a)$ is specified by the choice of a specific barotropic equation of state $P=P(\rho )$ and by the energy conservation equation $\dot{\rho }+3H(P+\rho ) =0$. Then, the Lagrangian (25) does not depend explicitly on the cosmic time *t* and the corresponding Hamiltonian is conserved:

26 $$\begin{aligned} \mathcal{H}=\frac{\partial L}{\partial \dot{a}} \, \dot{a} -L= 3a\dot{a}^2 -8\pi G a^3 \rho = C \,. \end{aligned}$$

The dynamics of general relativity is constrained dynamics: four of the field equations (specifically, the time-time and the time-space components of the Einstein field equations) are first order constraints [6, 7]. In the case of the FLRW geometry, due to the high degree of symmetry, the only constraint is the Hamiltonian constraint (time-time component), which imposes that $C=0$ and then (26) coincides with the Friedmann equation (16) [6].

### Symmetries

When there is a single term on the right-hand side of the cooling equation for the temperature difference $\theta \equiv T-T_{\infty }$, the analogous FLRW universe filled with a single perfect fluid, and ruled by the Friedmann equation (16), is spatially flat. In this case, the Einstein–Friedmann equations enjoy certain symmetries [10–12], which are studied in the cosmological literature, mostly in relation with solution-generating techniques [10–17]. In these symmetry transformations, one rescales time *t*, scale factor *a*, or Hubble function *H* and changes barotropic fluid appropriately, leaving the Einstein-Friedmann equations invariant.

The first symmetry [11] is

27 $$\begin{aligned} a\rightarrow & {} \tilde{a} = \frac{1}{a} \,, \end{aligned}$$

28 $$\begin{aligned} w\rightarrow & {} \tilde{w} = -(w+2) \,; \end{aligned}$$

it changes an expanding into a contracting universe and *vice-versa*. When translated to heat transfer, this symmetry is unphysical because the temperature difference $\theta$ must always be decreasing and this transformation turns it into an increasing quantity, corresponding to changing the sign of the right-hand side of the cooling model equations, therefore to a negative heat capacity—we will not consider it further.

The second symmetry [12] is

29 $$\begin{aligned}&a \rightarrow&\bar{a}=a^s \end{aligned}$$

30 $$\begin{aligned}&dt \rightarrow&d\bar{t} = s \, a^{ \frac{3(w+1)(s-1)}{2} } dt \end{aligned}$$

31 $$\begin{aligned}&\rho \rightarrow&\bar{\rho } = a^{ -3(w+1)(s-1)} \rho \,, \end{aligned}$$

where the real number $s\ne 0$ parametrizes the transformation. These symmetries form a one-parameter commutative group.

The third type of symmetry transformation [10] is

32 $$\begin{aligned} \rho\rightarrow & {} \bar{\rho }=\bar{\rho } (\rho ) \end{aligned}$$

33 $$\begin{aligned} H\rightarrow & {} \bar{H}=\sqrt{ \frac{ \bar{\rho }}{\rho } } \, \, H \end{aligned}$$

34 $$\begin{aligned} P\rightarrow & {} \bar{P }=-\bar{\rho } + \sqrt{ \frac{ \rho }{\bar{\rho }}} \, \left( P+\rho \right) \, \frac{ d\bar{\rho } }{d\rho } \,, \end{aligned}$$

where the function $\bar{\rho }(\rho )$ is positive and regular.

### Solutions in terms of elementary functions and roulettes

Methods to solve the Einstein–Friedmann equations (16)–(18) analytically and to study their phase space qualitatively are reviewed in [18–20]. New results are reported in Refs. [21–23], including the proof that the graphs of all solutions of the Friedmann equation (16) are roulettes [23]. A roulette is the trajectory (in two dimensions) of a point that lies on a curve rolling without slipping on another given curve. The most familiar example is probably the cycloid, which is the trajectory of a point on the rim of a bycicle wheel as the bycicle moves forward at constant speed on a horizontal surface and the wheel rolls (the graph of the corresponding solution, the scale factor of a $K=1$ FLRW universe filled with dust, appears in all cosmology and relativity textbooks [6–9]).

Finally, one wonders under which conditions it is possible to obtain analytical solutions of the Einstein-Friedmann equations in terms of elementary functions. For many choices of the cosmic matter filling the universe, by taking the square root of the Friedmann equation one obtains

35 $$\begin{aligned} \frac{da}{dt} = a F(a) \end{aligned}$$

where *F*(*a*) is a polynomial or a combination of powers. In many cases of physical interest, its integration is reduced to the task of computing an integral of the form [21]

36 $$\begin{aligned} I\left( x; p, q, r \right) = \int dx \, x^p \left( a+b \, x^r \right) ^q \,,\nonumber \\ p,q,r \in \mathbb {Q} \,, r\ne 0 \end{aligned}$$

(if $r=0$ the integral is trivial). This admits the hypergeometric function representation

37 $$\begin{aligned} I= & {} \frac{a^q x^{p+1} }{p+1} \, {}_2F_1\left( -q, \frac{p+1}{r}, \frac{p+r+1}{r}, -\frac{bx^r}{a} \right) +\text{ const. }\nonumber \\ \end{aligned}$$

which is, however, inconvenient for practical purposes. A necessary and sufficient condition for the integral (36) to be expressed in terms of elementary functions is the Chebysev theorem [24, 25]:

*the integral* (36) *admits a representation in terms of elementary functions if and only if at least one of*

$$\begin{aligned} \frac{p+1}{r} \,, \;\;\;\; q \,, \;\;\;\; \frac{p+1}{r} + q \end{aligned}$$

*is an integer.*

This theorem will be useful in the following.

## Cosmic analogues of cooling laws, Lagrangians, and symmetries

We now proceed to discuss the cosmological analogues of the various first order cooling laws of Sect. 1. The analogy provides Lagrangians $L=L\left( \theta , \dot{\theta } \right)$, symmetries, and mathematical properties of the solutions $\theta (t)$. The corresponding Euler–Lagrange equation

38 $$\begin{aligned} \frac{d}{dt} \left( \frac{\partial L}{\partial \dot{\theta }} \right) -\frac{\partial L}{\partial \theta }=0 \end{aligned}$$

admits a first integral which contains an arbitrary integration constant. Only one value of this constant reproduces the original cooling equation, as is common in the inverse variational problem of finding an action for a first order equation (see [26] for a detailed discussion).

### Newton cooling

Dividing Newton’s law of cooling (2) by $\theta$ and squaring yields

39 $$\begin{aligned} \left( \frac{1}{\theta } \, \frac{d\theta }{dt} \right) ^2 = k^2\,, \end{aligned}$$

which is formally the same as the Friedmann equation $H^2= \Lambda /3$ for a spatially flat, empty universe with positive cosmological constant $\Lambda =3k^2$. The solutions are expanding or contracting de Sitter universes with scale factors $a(t)= a_0 \, \text{ e}^{\pm kt}$, where only the negative sign applies to the analogy for both cooling/heating, giving $\theta (t)=\theta _0 \, \text{ e}^{-kt}$.

Inspired by the FLRW Lagrangian (25), one finds the effective Lagrangian for Newton’s law of cooling

40 $$\begin{aligned} L_1 \left( \theta , \dot{\theta } \right) = \dot{\theta }^2 +\left( \frac{ A h}{C} \right) ^2 \theta ^2 \,. \end{aligned}$$

Since $L_1$ does not depend explicitly on time, the corresponding Hamiltonian is conserved, $\mathcal{H}= \dot{\theta }^2 -k^2 \theta ^2=C$. Choosing $C=0$ and the negative sign in the square root of the resulting equation reproduces Newton’s law of cooling.

The symmetry  (29)–(31) is trivial in this highly symmetric situation: it merely changes the units of time $t\rightarrow \bar{t}=st$ and rescales the scale factor $a\rightarrow \bar{a}=a_0^s \, \text{ e}^{-k\bar{t}}$, leaving the density unchanged, $\bar{\rho }=\rho$.

The other symmetry is also trivial in this case. Since the cosmological constant is formally equivalent to a perfect fluid with a constant density $\rho _{(\Lambda )}=\Lambda / (8\pi G)$ and pressure $P_{(\Lambda )}=-\rho _{(\Lambda )}$, the transformation (32)-(34) reduces to the rescaling of $\Lambda$ and of the Hubble constant $\Lambda \rightarrow \bar{\Lambda }= \alpha \Lambda$, $H=\sqrt{\Lambda /3} \rightarrow \bar{H}=\sqrt{\bar{\Lambda }/3} = \sqrt{\alpha } H$.

### Dulong–Petit cooling

The Dulong–Petit cooling law (5) gives

41 $$\begin{aligned} \left( \frac{1}{\theta } \, \frac{d\theta }{dt} \right) ^2= g_0^2 \, \theta ^{2(n-1)} \end{aligned}$$

and the analogous Friedmann equation

42 $$\begin{aligned} H^2=\frac{8\pi G}{3} \, \rho \end{aligned}$$

describes again a spatially flat analogous universe, but this time the cosmological constant is zero and the cosmos is filled with a single perfect fluid with equation of state parameter

43 $$\begin{aligned} w= -\frac{(2n+1)}{3} \end{aligned}$$

and

44 $$\begin{aligned} \rho _0= \frac{3g_0^2}{8\pi G} \,. \end{aligned}$$

This is a phantom (i.e., $w<-1$) equation of state and this phantom fluid causes the universe to end in a Big Rip spacetime singularity at a finite future. Phantom fluids [27, 28] are very exotic forms of dark energy often invoked to explain the present acceleration of the cosmic expansion discovered in 1998 with type Ia supernovae [29], but are often favoured by cosmological observations. The characteristic feature of a phantom fluid is that it makes the universe that it fills expand so fast to explode at a finite time in a Big Rip singularity. While in the more familiar Big Bang or Big Crunch singularities [6–9] the scale factor *a*(*t*) vanishes, in a Big Rip it diverges instead. The scalar curvature invariants, $\rho$, and *P* diverge there. The expanding and contracting branches on either side of the Big Rip are disconnected because the spacetime stops at a curvature singularity, which does not belong to the spacetime manifold.

In our case, given the negative sign in the right-hand side of the cooling law, the analogous universe *contracts* from a Big Rip at time $t_0$ (where the curvature scalars, the energy density $\rho$ and the pressure *P* diverge) with scale factor

45 $$\begin{aligned} a(t)=\frac{a_0}{t-t_0} \end{aligned}$$

for $$t>t_0$$.

In seismology, the Omori–Utsu law [30, 31] giving the frequency $\dot{n}_s$ of the aftershocks following a main earthquake shock,

46 $$\begin{aligned} \dot{n}_{(s)} = -k n_{(s)}^p \,, \end{aligned}$$

obeys the same equation and has a Big Rip analogy [32]. Therefore, there is also an analogy between Dulong–Petit cooling and the “cooling” of active faults after a main shock. The pictorial expression “cooling of a seismically active zone” or “hot zone” acquires a precise meaning through this analogy. The dissipation of energy through secondary shocks becomes analogous to the removal of heat energy from a hot body by convection.

Similar power-law behaviour is ubiquitous in many sciences, including earth sciences, biology, population dynamics, computer science, economics, information theory, language, economy, and astronomy (e.g., [34]): for example, in hydrology the Brutsaert–Nieber law gives the recession flow in rivers as

47 $$\begin{aligned} \frac{dQ}{dt} = -k Q^{\alpha } \,, \end{aligned}$$

where *Q* is is the discharge at the river cross section at time *t* [33].

The Lagrangian corresponding to Dulong–Petit cooling is

48 $$\begin{aligned} L_2 \left( \theta , \dot{\theta } \right) = \theta \dot{\theta }^2 + g_0^2 \, \theta ^{2n+1} \,. \end{aligned}$$

Again, the Hamiltonian $\mathcal{H}= \theta \dot{\theta }^2 -g_0 ^2 \theta ^{2n+1}$ is conserved and choosing zero value for this “energy” and the negative sign in the square root reproduces the Dulong-Petit cooling law.

Another possible Lagrangian, which is explicitly time-dependent, is [35]

49 $$\begin{aligned} L_2 ' = \frac{1}{2} \left( c+t \right) ^{\frac{n}{n-1} } \dot{\theta }^2 \,; \end{aligned}$$

since $\partial L_2'/\partial \theta =0$, the momentum canonically conjugated to $\theta$ is conserved,

50 $$\begin{aligned} p_{\theta } \equiv \frac{\partial L_2'}{\partial \dot{\theta } } = \left( c+t\right) ^{\frac{n}{n-1} } \, \dot{\theta } = c_0 \,, \end{aligned}$$

where $c_0$ is an integration constant. This equation integrates to

51 $$\begin{aligned} \theta (t)= \frac{-c_0(n-1)}{ (c+t)^{\frac{1}{n-1}}} \,. \end{aligned}$$

Therefore,

52 $$\begin{aligned} \frac{1}{(c+t)^{\frac{1}{n-1}} } =\frac{\theta }{c_0 \left( 1-n \right) } \end{aligned}$$

which, substituted into Eq. (50), gives

53 $$\begin{aligned} \dot{\theta } = \frac{c_0^{1-n}}{ \left( 1-n \right) ^n } \, \theta ^n \equiv - g_0^2 \, \theta ^n \,. \end{aligned}$$

The symmetry (29)–(31) gives the new symmetry of the Dulong–Petit law

54 $$\begin{aligned} \theta\rightarrow & {} \bar{\theta }= \theta ^s \,, \end{aligned}$$

55 $$\begin{aligned} dt\rightarrow & {} d\bar{t} = s \, \theta ^{(1-n)(s-1)} dt \,. \end{aligned}$$

This particular scaling invariance may be useful when scaling considerations are needed in applications (for example to scale a small system in the lab to industrial size).

Let us consider now the other symmetry (32)–(34): in order to preserve the analogy, the equation of state must be preserved with $\bar{w}=-\left( 2\bar{n}+1\right) /3$, which leads to

56 $$\begin{aligned} \left( \frac{\bar{\rho }}{\rho } \right) ^{-3/2} \, \frac{d\bar{\rho }}{d\rho } = \frac{ \bar{w}+1}{w+1} \,, \end{aligned}$$

or

57 $$\begin{aligned} d\left( \bar{\rho }^{-1/2} \right) = \frac{ \bar{w}+1}{w+1} \, d\left( \rho ^{-1/2} \right) \,. \end{aligned}$$

The solution is

58 $$\begin{aligned} \bar{\rho } = \left( \frac{w+1}{\bar{w}+1} \right) ^2 \rho = \left( \frac{n-1}{\bar{n}-1} \right) ^2 \rho \,, \end{aligned}$$

which implies $\bar{\rho }_0 = \left( \frac{n-1}{\bar{n}-1} \right) ^2 \rho _0$, or

59 $$\begin{aligned} \bar{g}_0= \frac{n-1}{\bar{n}-1} \, g_0 \,. \end{aligned}$$

Equation (31) then gives

60 $$\begin{aligned} \bar{H}=\frac{n-1}{\bar{n}-1} \, H \end{aligned}$$

and

61 $$\begin{aligned} \bar{a}(t)=\bar{a}_0 \, t^{ \frac{n-1}{(1-n)(\bar{n}-1)}} = \left[ a(t)\right] ^{ \frac{ n-1}{\bar{n}-1} } \,. \end{aligned}$$

Therefore, the transformation for the temperature difference is

62 $$\begin{aligned} \theta \rightarrow \bar{\theta }=\theta ^{ \frac{ n-1}{\bar{n}-1} }\,. \end{aligned}$$

### Approximated Newton–Stefan cooling

Dividing by $\theta$ and squaring, the approximated Newton–Stefan cooling law (13) becomes

63 $$\begin{aligned} \left( \frac{1}{\theta } \, \frac{d\theta }{dt}\right) ^2 = \alpha ^2 + 2 \alpha \beta \, \theta + \beta ^2 \theta ^2 \,, \end{aligned}$$

which is analogous to the Friedmann equation (16) for a universe with flat spatial sections, cosmological constant $\Lambda = 3\alpha ^2$, and two perfect fluids with energy densities

64 $$\begin{aligned} \rho _{(1)}= & {} \frac{3 \alpha \beta }{4\pi G } \, a \end{aligned}$$

65 $$\begin{aligned} \rho _{(2)}= & {} \frac{3\beta ^2}{8\pi G } \, a^2 \,, \end{aligned}$$

and equation of state parameters $w_{(1)}= -4/3 \,, w_{(2)}= -5/3$. These are both phantom fluids. Since $\dot{a}<0$ is the only possibility in this analogy, they concur with the cosmological constant to make the analogous universe contract. The exact solution (14) is asymptotic to

66 $$\begin{aligned} a(t) \simeq \sqrt{\frac{3}{\Lambda }} \, \, \frac{ \theta _0 \, \text{ e}^{-\sqrt{\Lambda /3} \, t}}{ \sqrt{\Lambda /3} +\beta \theta _0} \end{aligned}$$

as $t\rightarrow +\infty$, which is explained by noting that the energy densities of the phantom fluids (which scale as the decreasing *a* or $a^2$, respectively) decay, while the energy density of the cosmological constant $\rho _{(\Lambda )}=\Lambda /(8\pi G)$ remains constant and comes to dominate over the other two as the universe evolves. In other words, the contracting de Sitter space is an attractor in the phase space of the solutions of the approximated Newton-Stefan model. In fact, it is an attractor in the phase space of the *exact* Newton-Stefan model since higher order terms in $\theta$ in the right-hand side of Eq. (8) become negligible sooner than the lower order terms in $\theta$ and $\theta ^2$ that were retained in the approximation (13).

The Lagrangian is now

67 $$\begin{aligned} L_3 \left( \theta , \dot{\theta } \right) = \theta \dot{\theta }^2 + \theta ^3 \left( \alpha + \beta \theta \right) ^2 \,. \end{aligned}$$

The special case $\beta =0$ generates the Lagrangian $L_3 \Big |_{\beta =0}= \theta L_1$ equivalent to (40) for Newton cooling, while setting $\alpha =0$ reproduces exactly the Lagrangian (48) for $n=2$ Dulong-Petit cooling.

Since $L_3$ does not depend explicitly on the time *t*, the corresponding Hamiltonian is conserved,

68 $$\begin{aligned} \mathcal{H}=\frac{\partial L_3}{\partial \dot{\theta }} \, \dot{\theta } - L_3 = \theta \, \dot{\theta }^2 -\theta ^3 \left( \alpha +\beta \theta \right) ^2 = C_0 \,, \end{aligned}$$

where $C_0$ is an integration constant, or

69 $$\begin{aligned} \left( \frac{1}{\theta } \, \frac{d\theta }{dt} \right) ^2 = \left( \alpha + \beta \theta \right) ^2 + \frac{C_0}{\theta ^3} \,. \end{aligned}$$

By choosing $C_0=0$, one obtains $\dot{\theta }= \pm \left( \alpha \theta +\beta \theta ^2 \right)$. Only the lower sign reproduces the original problem, as discussed in [26].

Since there are two perfect fluids plus the cosmological constant, the symmetries (29)–(34) valid for $K=0$ and a single fluid do not apply here.

If the coefficients are allowed to change signs, the equation describing the truncated Newton–Stefan model appears in other field of physics and mathematics. For example, the logistic equation can be reproduced or, in a simplified laser emission model, the photon emission rate *dn*/*dt* is related to the number of photons *n*(*t*) in an excited state by [36, 37]

70 $$\begin{aligned} \frac{dn}{dt} = -\alpha n -\beta n^2 \,. \end{aligned}$$

### More general models

More general models of convective-radiative cooling, which include the exact Newton–Stefan cooling (8) are given by

71 $$\begin{aligned} \frac{d\theta }{dt} = - \theta ^p \left( \alpha +\beta \theta ^r\right) ^n \end{aligned}$$

where *n* is a positive integer. The integration of this equation reduces to computing the integral

72 $$\begin{aligned} \int d\theta \, \theta ^{-p} \left( \alpha +\beta \theta ^r \right) ^{-n} \,, \end{aligned}$$

which is of the form (36) provided that *r* is rational. In practice, since no thermal physics experiment is sufficiently precise to distinguish between a real number *r* and its rational approximation, *r* can always be chosen to be rational. Since here *q* is the integer $-n$, the Chebysev theorem applies and one concludes that the solution of Eq. (71) can always be expressed in terms of elementary functions. More precisely, the left-hand side of the equation

73 $$\begin{aligned} \int d\theta \, \theta ^{-p} \left( \alpha +\beta \theta ^r \right) ^{-n} =-\left( t-t_0 \right) \,, \end{aligned}$$

where $t_0$ is an integration constant, can be reduced to elementary functions. This does not guarantee that this $t(\theta )$ relation can be inverted explicitly to provide $\theta (t)$, but in many situations this is immaterial. For example, for $p=2, r=1, n=2$, one obtains

74 $$\begin{aligned} \frac{\alpha \beta }{\alpha +\beta \, \theta } +\frac{\alpha }{\theta } +2\beta \ln \left( \frac{\theta }{\alpha +\beta \, \theta }\right) =\alpha ^3 \left( t-t_0\right) \,. \end{aligned}$$

In all models of cooling/heating for which the cosmological analogy is valid, the solution $\theta (t)$ has a graph that is a roulette, as is the case for the corresponding analogous universes [21].

## Conclusions

Formal analogies between FLRW cosmology and various models of convective-radiative heating/cooling exist. In principle, for an equation of the type $\dot{\theta }=\theta f(\theta )$, one can also draw a mechanical analogy with the motion of a point particle in one dimension subject to an appropriate conservative force (see Ref. [26]), but the cosmic analogy is much more interesting.

In nature, temperature gradients tend to be smoothed out and disappear once final states of thermal equilibrium are reached, unless these gradients are maintained by steady heat sources. As is well known, this process identifies an arrow of time intrinsic to macroscopic objects composed of many particles or subsystems, although a preferred time direction does not exist in the equations of fundamental microscopic physics, which are time-reversible. In the analogy between the various phenomenological models of thermal physics discussed above and the Friedmann equation, the analogue of the thermodynamical arrow of time is the formal cosmological arrow of time (“formal” because the real universe expands instead of contracting). All comoving objects in these fictitious universes are dragged by the cosmic contraction and their proper distances eventually reduce to nothing as the scale factor *a*(*t*) vanishes asymptotically at late times, just as the temperature differences $\theta$ disappear in both convective-radiative heating and cooling.

Specifically, we have discussed models of heating / cooling in which the function $f^2(\theta )$ appearing in Eq. (22) is a polynomial of power laws or inverse power laws. More general forms of $f(\theta )$ can also be studied. In this case, imposing the energy conservation equation (18) leads to a nonlinear barotropic equation of state $P=P(\rho )$ for the analogous cosmic fluid. Such equations of state have been studied in cosmology, particularly in the last decade in relation with hypothetical forms of exotic dark energy [38–46]. Equations of state of the cosmic fluid of the form $P = \sum _{k=1}^m c_k \rho _{(k)}^k$ were studied in Refs. [22, 23, 49–51]. They give rise to a cosmic analogy provided that the energy conservation equation is satified, which amounts to

75 $$\begin{aligned} \int \frac{d\rho }{ \sum _{k=1}^m c_k \rho _{(k)}^k +\rho } = -3\ln a \,. \end{aligned}$$

The special case of a quadratic equation of state has been scrutinized more closely [47, 48, 52–55], while pressures depending on fractional powers of the density were studied in [43]. Thus far, these equations of state are completely speculative compared to well established linear ones.

We have shown that, for all models of cooling/heating for which the formal analogy with the Friedmann equation holds (including the new models (71)), the solutions are roulettes. Moreover, in all situations of practical interest considered here, the analytical solutions can be expressed in terms of elementary functions, and new symmetries have been derived. To the best of our knowledge, these mathematical properties of the most popular cooling models were not discussed in previous literature and emerge due to recent results in cosmology [21] and to the analogy with the Friedmann equation.

## Data Availability

This manuscript has no associated data or the data will not be deposited. [Authors’ comment: This is theoretical work with no associated data.]

## Footnotes

1 Since we are going to include the Stefan–Boltzmann law in the discussion, *T* is taken to be the absolute temperature, although this is not necessary for convective cooling.

2 We adopt the notation of Refs. [6, 7], in which units are used such that the speed of light is unity, while *G* is Newton’s constant.
